# <mds_ies_db>: a database of ciliate genome rearrangements

**DOI:** 10.1093/nar/gkv1190

**Published:** 2015-11-19

**Authors:** Jonathan Burns, Denys Kukushkin, Kelsi Lindblad, Xiao Chen, Nataša Jonoska, Laura F. Landweber

**Affiliations:** 1 Department of Ecology and Evolutionary Biology, Princeton University, NJ 08544, USA; 2 Department of Mathematics & Statistics, University of South Florida, FL 33620, USA

## Abstract

Ciliated protists exhibit nuclear dimorphism through the presence of somatic macronuclei (MAC) and germline micronuclei (MIC). In some ciliates, DNA from precursor segments in the MIC genome rearranges to form transcriptionally active genes in the mature MAC genome, making these ciliates model organisms to study the process of somatic genome rearrangement. Similar broad scale, somatic rearrangement events occur in many eukaryotic cells and tumors. The <mds_ies_db> (http://oxytricha.princeton.edu/mds_ies_db) is a database of genome recombination and rearrangement annotations, and it provides tools for visualization and comparative analysis of precursor and product genomes. The database currently contains annotations for two completely sequenced ciliate genomes: *Oxytricha trifallax* and *Tetrahymena thermophila*.

## INTRODUCTION

Ciliated protists are microbial eukaryotes that use cilia for locomotion and contain two types of nuclei within their cytoplasm: a somatic macronucleus (MAC)—which provides templates for the transcription of all genes required for asexual growth, and a germline micronucleus (MIC)—used for the exchange of meiotic products during sexual reproduction.

During conjugation (sexual reproduction), haploid gametic nuclei exchange between pairs of mating cells to form a diploid zygotic nucleus, a copy of which develops into a new MIC and MAC. DNA in the MIC remains organized in large chromosomes. In contrast, the much smaller chromosomes in the MAC genome form via extensive fragmentation, elimination and sometimes broader rearrangement of germline DNA, coupled to DNA amplification and telomere addition (1). This process produces a set of over 16 000 small acentric MAC chromosomes in *Oxytricha* (2) and 181 in *Tetrahymena* (3).

The extent of genome reorganization varies greatly among ciliate species. In ciliates belonging to the class Spirotrichea (which includes *Oxytricha trifallax*), the level of DNA processing in the formation of a new MAC is extraordinary: the original zygotic chromosomes are fragmented into over 225 000 precursor DNA pieces, called *Macronuclear-Destined Sequences* (MDSs), with accompanying loss of approximately 90% of the DNA complexity (4). The resulting MAC chromosomes, are amplified to thousands of copies each (1). In *Oxytricha*, approximately 90% of MAC chromosomes encode a single gene, flanked at the 5′ and 3′ ends by very short (average 50 bp) untranslated regions plus telomeres (2). The size of these molecules ranges from ≈0.31 to 66 kb (2).

**Figure 1.**
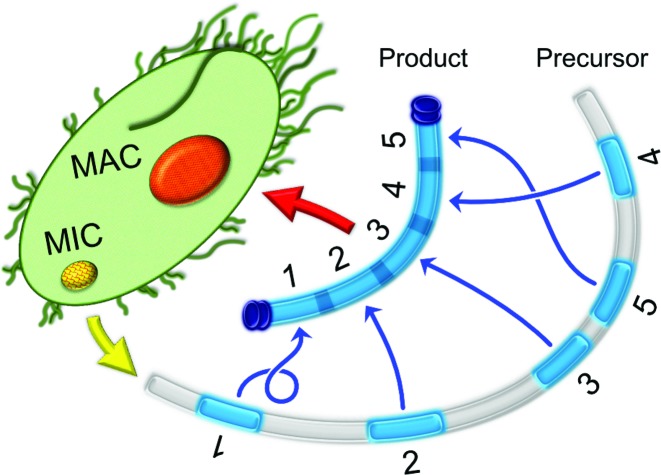
In the somatic macronucleus (MAC), chromosomes assemble from precursor MDS building blocks (blue), which may be scrambled in some species. In the germline micronucleus (MIC), the Macronuclear-Destined Sequences (MDSs) for all somatic chromosomes are dispersed over the long chromosome, and interrupted by *Internally-Eliminated Sequences* (IESs) and other noncoding DNA (gray), that may include transposons. In species such as *Oxytricha*, an MDS may appear in a permuted order, or inverted in the precursor relative to the final order expressed in the product version.

In all ciliates, AT-rich *Internally-Eliminated Sequences* (IESs) interrupt precursor MDSs (see Figure 1). While the IESs in *Tetrahymena* mostly fall between genes, with few exceptions (5), the IESs in *Oxytricha* and *Paramecium* frequently interrupt genes. Furthermore, the complex IESs in *Oxytricha* can even contain MDSs for other genes or entire genes themselves (4). Furthermore, approximately 20% of *Oxytricha*'s macronuclear genes contain MDSs that are present in a permuted order or orientation (4). These MDSs rearrange during MAC development according to long RNA templates as guides (6). This added layer allows *Oxytricha* to rebuild its functional somatic chromosomes from a highly scrambled genome (see Figure 1).

The last few nucleotides of each MDS are usually repeated at the beginning of the next consecutive MDS. In *Oxytricha* and related species, these junction sequence repeats are called *pointers*, and recombination between these 2–20 bp direct repeats leaves precisely one copy in the macronucleus. Except for the longest pointers, however, these short sequences are usually present in multiple locations in the precursor MIC gene loci (7). Hence, this underscores the need in *Oxytricha* for an RNA-guided, error-correcting mechanism, experimentally demonstrated in (6), to accurately establish and maintain wild-type versions of somatic genes across generations.

For a more thorough review of our current knowledge of the mechanism of RNA-guided DNA rearrangement and DNA descrambling in the ciliate *Oxytricha*, see (8).

### Rearrangement annotations

Annotated sequence elements consist of the recombination building blocks: MDSs, IESs and pointers. The rearrangement maps conceptually describe how each organism deconstructs its micronuclear genome into thousands of MDSs, and then reassembles the pieces correctly for the next generation's macronucleus.

Specifically, a rearrangement map lists the precursor order and orientation of each MDS in a micronuclear contig, relative to the orthodox order and orientation of MDSs in the product MAC contig. In Figure 1, the rearrangement map is $\overline{M}_1 M_2 M_3 M_5 M_4$ where the bar in $\overline{M}_1$ indicates that the orientation of MDS 1 is reversed relative to the other MDSs in the macronucleus. A map is *scrambled* if the precursor order or orientation of one or more MDSs differs from the product version.

Before complete genome sequences were available for both nuclei, most rearrangement maps were limited to surveys of single genes (9), since recombination annotations require knowledge of both the precursor and product versions. With the advent of new sequencing technologies, there have been major advancements in the sequencing of ciliate genomes: reference *O. trifallax* macronuclear (2) and micronuclear (4) genome assemblies were both reported in the past three years. The *T. thermophila* macronuclear genome (3,10) was published in 2006, and the Broad Institute of Harvard and MIT (http://www.broadinstitute.org) recently sequenced the *Tetrahymena* micronuclear genome as part of the *Tetrahymena* Comparative Sequencing Project.

Annotations for *O. trifallax* and *T. thermophila* were generated using the program MDS/IES DNA Annotation Software (available at http://knot.math.usf.edu/midas/). This application first masks the telomeric sequences in the macronuclear genome assembly, uses BLAST to find high scoring pairs between the precursor and product genomes, and then searches for a consensus among the pairs to annotate an MDS. After all the MDS regions have been identified, the program matches each precursor MDS with its corresponding locus in the product genome, while recording the relative precursor-to-product order and orientation. Finally, the program outputs the rearrangement maps and annotations for the telomeric regions, MDS precursor and product genomic regions, and other high scoring pairs that may be either allelic, paralogous or degenerate copies of former MDSs. The overlapping MDS regions in the product genome can be interpreted as the pointer sequences, and the intervening regions between MDSs in the product genome comprise the IESs, which may contain transposable elements and other repetitive AT-rich DNA.

For further details about the MDS/IES DNA Annotation Software algorithm, see http://knot.math.usf.edu/midas/algorithm.html.

In the *Oxytricha* data, the MDS regions associated with a product contig may be spread across more than one precursor contig. Table 1 indicates that two or more rearrangement maps can be associated with a 2-telomere MAC contig. This may be due to discontinuities in the genome assembly of the precursor MIC locus for a MAC contig. Such instances can result from the presence of either very long IESs (and hence very long precursor MIC loci that map to more than one MIC contig) or the possibility that some MAC genes might explicitly require MDSs from more than one MIC locus (7). Alternatively, the presence of both alleles and paralogous MDSs in the precursor genome can inflate the number of rearrangement maps for a given locus.

**Table 1.** Representative summary of the data in the database for *Oxytricha*

MAC MDSs	298 041
MIC MDSs	752 901^a^
2-Telomere contigs	17 198^b^
Rearrangement maps^c^	39 128
Complete rearrangement maps^d^	15 210
Scrambled rearrangement maps^c^	5 909
Scrambled complete maps^d^	1 548

^a^Includes paralogous MDSs and alleles.

^b^Data obtained from (2) and unpublished data from K.L., X.C and L.F.L.

^c^Maps from MAC contigs with 3′ and 5′ telomeres that are ≥30% covered by their associated MIC contig.

^d^Maps that contain all of the MDSs of the MAC contig.

## DATABASE DESCRIPTION

The <mds_ies_db> is unique among genetic databases, because it focuses on comparing and contrasting the precursor/product pairs of ciliate germline and somatic genomes. Several recent cancer genome projects also compare and contrast somatic versus reference germline genomes (11–14), but unlike cancer cells the ciliate genome rearrangements are faithfully programmed across generations. This permits a high level of reproducibility and resolution that can provide benchmark standards for other methods that measure somatic rearrangement.

Database features include the rearrangement annotations and locations of germline-limited or MAC-specific genes. This complements other existing ciliate databases that concentrate on genome assemblies, gene lists and protein domains, such as the TGD (10) (http://ciliate.org), TCD (http://broadinstitute.org), OxyDB (15) (http://oxy.ciliate.org) and ParameciumDB (16) (paramecium.cgm.cnrs-gif.fr), or gene function and expression, e.g. TFGD (17) (http://tfgd.ihb.ac.cn) and TGED (18) (http://tged.ihb.ac.cn). Moreover, the <mds_ies_db> is expandable and updatable, and ready to house the rearrangement annotations for other sequencing projects as more reference genomes or new genome releases and updates become available.

### Searches

Both quick navigation-bar and advanced-form searches are present to facilitate data retrieval. The main navigation bar includes quick searches for gene, contig and sequence ID numbers. There are currently three advanced-form searches: Contig, Gene and Sequence searches. The *Contig Search* (shown in Figure 3) filters contigs by organism, nucleus type, sequence length, number of genes and the number of contigs; *Gene Search* filters genes by organism, nucleus type, description, domains and restriction to either the macronucleus or micronucleus, and the *Sequence Search* allows the user to BLAST a nucleotide or protein sequence against the genomes and proteomes of the organisms in the database. Sequences can either be input manually, or files can be dragged-and-dropped into the input text area.

**Figure 2.**
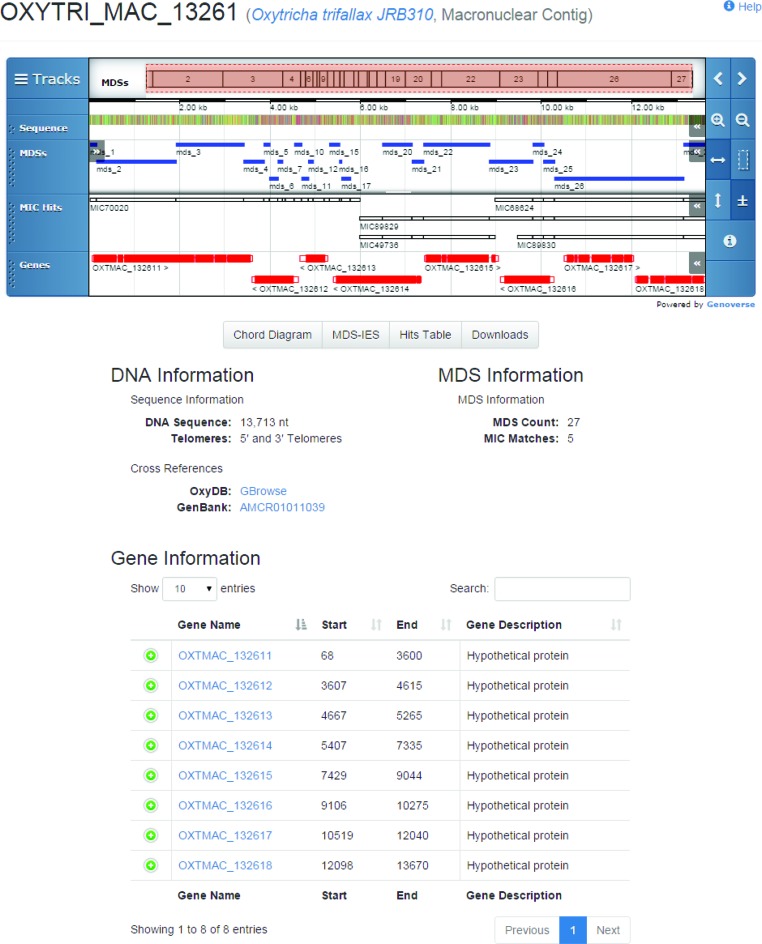
Each macronuclear and micronuclear contig has a display page that includes (i) the contig name and aliases, (ii) a genome browser, (iii) information sections for the contig, MDSs and genes, and (iv) links to the corresponding chord diagram, MDS-IES, hits information table and download pages. The genome browser displays annotations for the nucleotide sequence, MDS annotation, HSPs with corresponding contigs and the gene annotations for the contig.

All advanced-form searches return links to the matching contig and gene display page (see Figure 2), consisting of the name and alias of the contig and genes, a genome browser containing the contig's genome sequence and associated annotations, links to the matching chord diagram, MDS–IES table of annotation, a table of high scoring pairs to other MAC and MIC contigs, and sections containing the contig's *DNA Information*, *MDS Information* and *Gene Information*.

**Figure 3.**
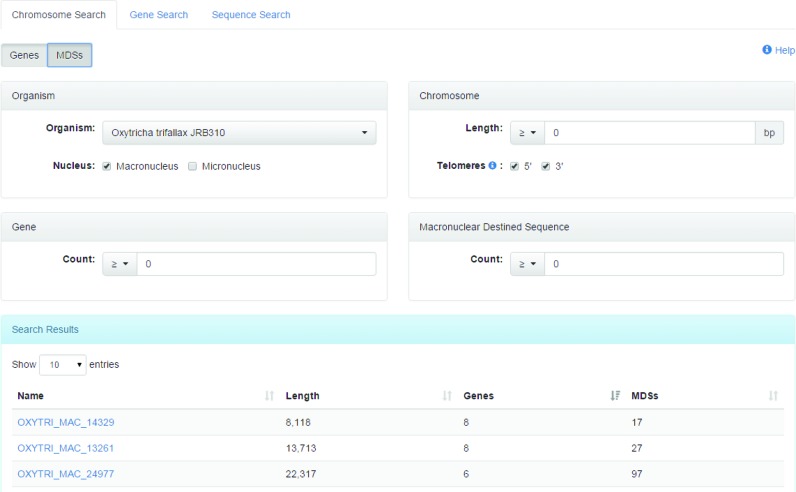
Screenshot of the Contig Search form that allows the user to search for specific contigs, and to filter the results by the organism name, nucleus type, sequence length, presence of telomeres, number of genes and number of MDSs.

### Genome browser

The <mds_ies_db> uses Genoverse (http://genoverse.org), a native HTML5 genome browser, to display a contig's nucleotide sequence, MDS annotation, hits to other MAC or MIC contigs and gene annotations (see Figure 2) on separate tracks. The browser features dynamic zooming and scrolling of the tracks, and it is possible for a user to add their own tracks by dragging-and-dropping an XML, JSON, GFF, GFF3 or BED file into the browser.

### Chord diagrams

**Figure 4.**
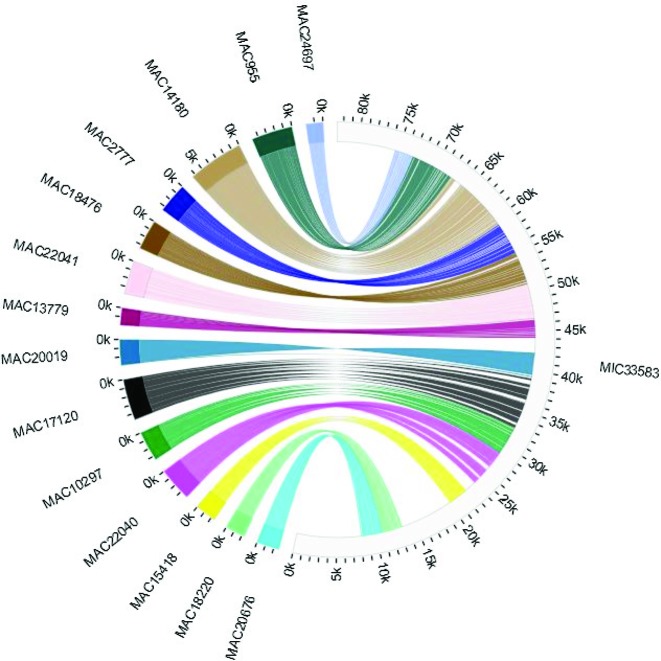
Chord diagram display is one of the database tools to visually represent matching regions of MIC and MAC DNA sequences. This figure depicts matching high scoring pairs between OXYTRI_MIC_33583 in the germline and several MAC contigs, whose precursor sequences are distributed across OXYTRI_MIC_33583.

Displaying matches between repetitive regions on one contig to a single location on another contig is not convenient for a single track browser. Chord diagrams (a.k.a Circos Plot) allow a user to easily visualize any arrangement map between two sequences with corresponding loci. The <mds_ies_db> uses the D3 Javascript library to render scale diagrams of the high scoring pairs from a macronuclear contig to its related micronuclear contigs and vice versa. In the example in Figure 4, MIC contigs are colored gray and each MAC contig is assigned a unique color. Each HSP is colored to match its associated MAC contig.

### MDS–IES and hits tables

**Figure 5.**
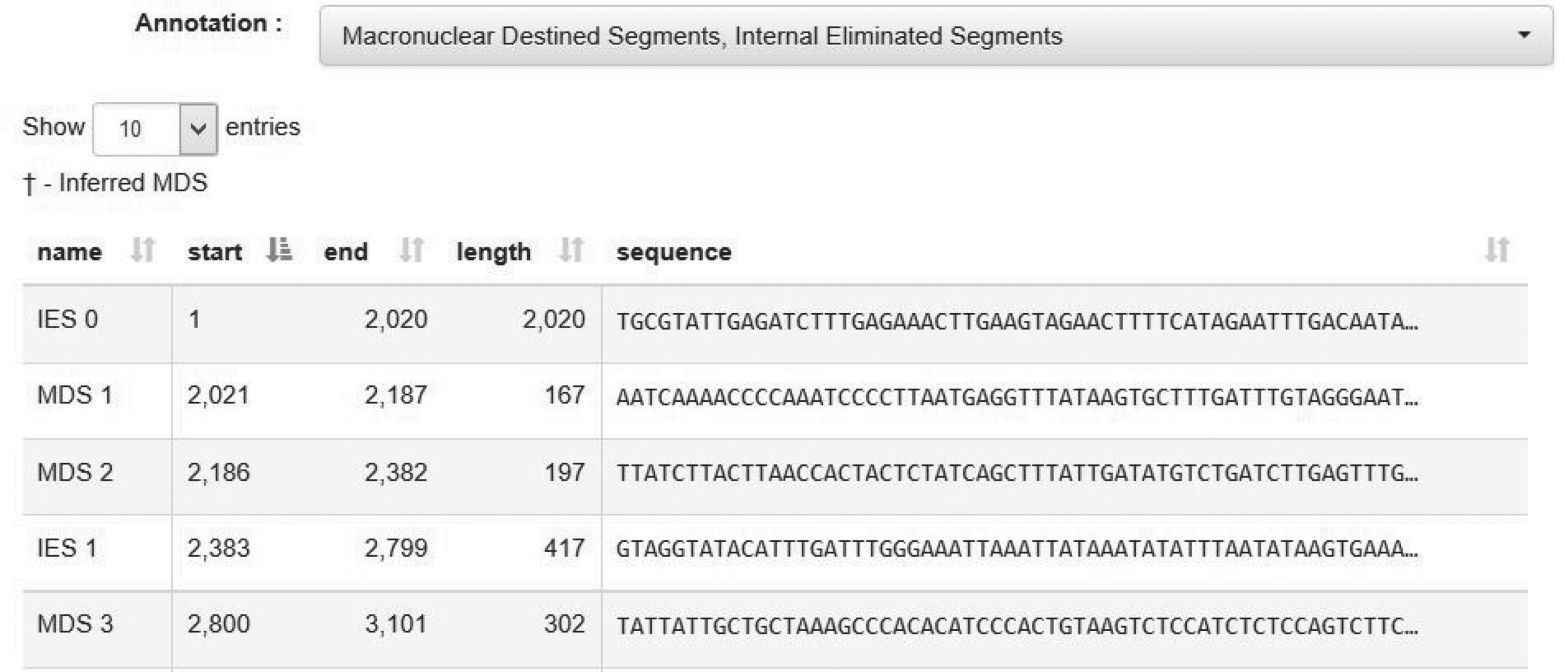
Screenshot of the MDS–IES table. The annotation dropdown menu allows the user to filter for MDS, IES and/or pointers. Every truncated sequence can be clicked to reveal the full sequence in another window.

Each contig display page contains buttons that activate pop-up tables for MDS, IES, and pointer annotations and hits to other contigs. The *MDS–IES Table* has filters to show the annotations and sequences for any combination of MDSs, IESs and pointers (see Figure 5). Sequences that are too long to fit within the table are truncated, but clicking on the sequence will open a new window with the full sequence. When a macronuclear contig is not fully covered by sequences in the micronucleus, this leads to one or more gaps or *missing MDSs*, in the annotation for the macronuclear version of the gene. Both MDSs and missing MDSs (annotated separately) are included in the MDS–IES Table.

**Figure 6.**
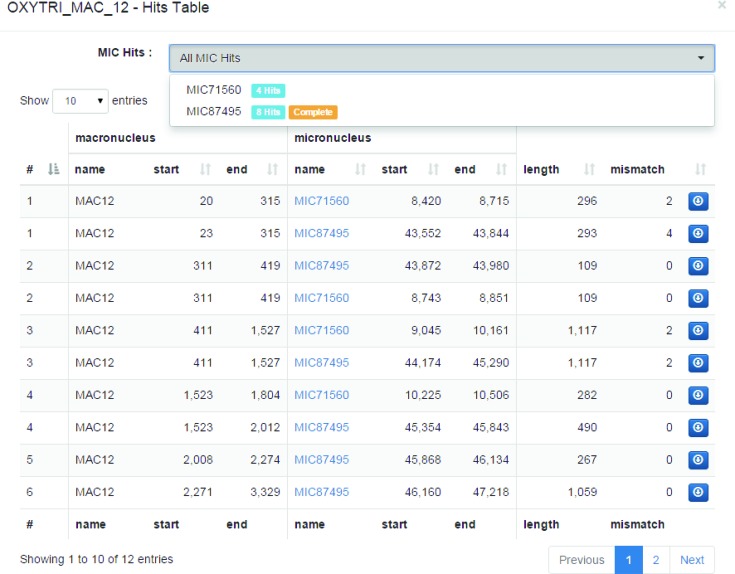
Screenshot of the MDS–IES table display. The hits dropdown menu allows the user to filter for specific contigs. Clicking the button at the end of the rows opens up another window that shows the MAC and MIC version of the sequence with the mismatches highlighted.

Similarly the *Hits Table* contains a list of high scoring pairs to other contigs, with filters to isolate matches between specified contigs (see Figure 6).

### Downloads

Customized downloads are available for each contig, which may include any combination of the contig's (i) nucleotide and protein sequences as .fasta files, (ii) annotations for telomere, MDS, IES, pointers, genes and domains as .gff3 files, and (iii) RNA-seq expression and rearrangement map in either .csv or Excel spreadsheet format.

### Cross-references to external databases

The *DNA Information* and *Gene Information* sections of the contig display page contain cross-references to the corresponding entries in TGD (10) (http://ciliate.org), OxyDB (15) (http://oxy.ciliate.org) and GenBank (19) (http://www.ncbi.nlm.nih.gov/).

The *Sources and Citations* section, located in Data dropdown of the main navigation menu, contains links and citations for the reference genome sequences of *O. trifallax* (2,4) and *T. thermophila* (10,15).

### Database architecture

The <mds_ies_db>, a modernization and expansion of the MDS_IES_DB (9), is built with the MySQL database management system version 5.6.25, and hosted using Apache on a LINUX server. The main user interface of the new database is built as an HTML5 website using the Bootstrap, D3 and DataTables JavaScript libraries. The database also interfaces with wwwblast as a part of the built-in nucleotide and protein sequence search. The current database is approximately 12 GB, and consists of more than 20 tables with over 75 million rows.

## AVAILABILITY

The information contained in the <mds_ies_db> is free and open to the public, and can be found at http://oxytricha.princeton.edu/mds_ies_db. Since the database was designed using the Bootstrap framework, the website is responsive to a variety of resolutions, making it desktop, tablet and mobile friendly. All of the dynamic, interactive features of the database are written in Javascript, so users may fully utilize the website without downloading any external software or installing browser add-ons.

The Downloads page, under the Data navigation dropdown in the main navigation menu, offers bulk download links for all MAC and MIC genome assemblies and annotations for MDS, IES and pointer sequences.

The manual for the database is located under the *Help* tab in the main navigation bar, and provides descriptions for the built-in searches and extended information about the database, sequence naming conventions, and genome display tools and features.
